# The Lord Mayor and "Hospital Saturday"

**Published:** 1912-10-12

**Authors:** Thos. Boor Crosby

**Affiliations:** Lord Mayor. Mansion House, London, E.C.


### The Editor's Letter-Box.

### The Lord Mayor and "Hospital Saturday."

*To the Editor of* The Hospital.

Sir,—As a member of the medical profession and President for the year of the Hospital Saturday Fund, I have much pleasure in pleading the cause of "Hospital Saturday," which falls on the 12th inst.

Since the discontinuance of the annual street collection in 1897, this well-managed Fund has progressed uninterruptedly and done excellent work both for the participating institutions and its general supporters. In all its doings the principles of self-help and mutual help are insisted upon. Last year its income amounted to £36,932, whilst a further sum of £8,536 was received by the Distribution, Surgical Appliance, and Ambulance Committees in part payment of the cost of benefits supplied, so that the total was £45,468. This year, probably owing to temporary causes, the rate of progress has slightly fallen, and it is necessary to appeal most earnestly for help on "Hospital Saturday." There must be many who, knowing the great work done by the Metropolitan hospitals and having enjoyed the blessings of health, will be willing to aid those less fortunate than themselves. There may be others, now restored to health through the ministration of the medical profession, who may be glad to send a thankoffering. Donations may be sent to me at the Mansion House, or to the Treasurer of the Fund, the Hon. H. L. W. Lawson, M.P., 54 Gray's Inn Road, London, W.C.—I am, Sir, your obedient servant,

Thos. Boor Crosby, Lord Mayor.

Mansion House, London, E.C., October 10.

